# 

**Published:** 1923-04

**Authors:** 


### HOSPITAL and HEALTH

REVIEW

*Established 1886* No. 1861. *Vol. LXXII.*

*New Series.* Vol. II. No. 19 *April* 1923 Price Sixpence

CONTENTS.

| | PAGE |
|---|---|
| The New Housing Policy | 157 |
| Future of the Voluntary Hospitals | 158 |
| Notes and Comments | 159 |
| The Hospital and Nursing Exhibition | 163 |
| Insurance Scraps of Paper. By R. W. Harris | 164 |
| Voluntary Hospitals Commission | 165 |
| Public Health Services in Southern Ireland | 166 |
| The Public Health: Interviews with Local Authorities—Middlesex | 167 |
| The Blind Man at Work | 168 |
| Sussex County Hospital | 169 |
| Insurance Notes | 170 |
| Hospital Progress and Finance | 171 |
| Ambulance Work in Bombay | 173 |
| Hygiene and Nursing in Belgium | 173 |
| Replanting the Black Country | 174 |
| Mental Treatment | 177 |
| An Open-Air School | 177 |
| Reviews | 178 |
| Correspondence | 181 |
| The Romance of Cod-Liver Oil | 182 |
| Echoes of the Month | 183 |
| Public Health in Parliament | 184 |
| Hospital and Health News | 185 |
| Recent Appointments | 186 |
| The Hospital and Nursing Exhibition: Some of the Stalls | 187 |

